# Ohio College of Dental Surgery

**Published:** 1881-04

**Authors:** 


### EDITORIAL.

### OHIO COLLEGE OF DENTAL SURGERY.

The Thirty-fifth Annual Commencement Exercises of the Ohio College of Dental Surgery took place at College Hall, on Thursday evening, March 3, 1881. The valedictory address was delivered by Rev. Nathaniel West, D. D.; the class oration by H. D. Eggers, D. D. S.

The degree of D. D. S. was conferred on the following members of the graduating class by Geo. W. Keely, D. D. S., President of the Board of Trustees.

| | | | |
|---|---|---|---|
| C. S. Archer | Ind. | W. V. Grove | N. Y. |
| P. D. Anderson | Ky. | B. P. Ingram | O. |
| D. A. Askew | Miss. | E. L. Jauncey | Ill. |
| E. S. Bowen | Mass. | G. S. Junkerman | O. |
| H. W. Brodbeck | Ind. | W. C. Jeffry | Ind. |
| E. P. Binford | Ind. | Julius Klethke | Prussia |
| O. Buckwalter | O. | J. D. Morris | O. |

| | | | |
|---|---|---|---|
| J. S. Converse | O. | S. H. Millikin | O. |
| S. B. Cook | Tenn. | J. B. McGee | Ky. |
| C. C. Corbett | Vt. | Zeno F. Meyer | Wis. |
| T. Y. Cooper | Ky. | S. D. Myers | O. |
| F. H. Deterding | Pa. | C. B. Orbison | O. |
| John Donovan | O. | W. G. Price | Ind. |
| H. D. Eggers | Ky. | Edward Pitwood | Ill. |
| T. H. Foulds | O. | E. H. Rothe | O. |
| M. L. Hughes | Ky. | J. E. Robinson | O. |
| H. O. Howe | O. | B. G. Rees | Ky. |
| J. C. Herron | Ind. | E. C. Sears | O. |
| C. E. Hale | Minn. | R. S. Taylor | O. |
| E. H. Hawkins | O. | | |

The Dean announced the award of Prizes for the Session, as follows:

I. A prize of a Dental Engine by the Faculty, for the best general examination. Thos. H. Foulds, Jr., O.

II. A prize of a set of Plugging Instruments by Prof. H. A. Smith, for the best specimen of gold fillings in the mouth.
S. B. Cook, Tenn.,
with honorable mention of C. E. Hale and E. P. Binford.

III. A prize of a Class Microscope by Prof. Clayton, for the best essay on Microscopical Anatomy of Teeth.
Thos. H. Foulds, Jr., O.

IV. A prize of a Dental Case by Prof. Rawls, for the best essay on General Inflammation and treatment.
John Donovan, O.

V. A prize of a complete Farrar's Alveolar Abscess Syringe, by Prof. Taylor, for the best essay on Dental Hygiene.
H. O. Howe, O.

VI. A prize of a Gold Medal by Prof. Cassidy, for the best essay on Oral Chemistry. G. S. Junkerman, O.

VII. A prize by Geo. W. Fells and Bro., of an S. S. White Bracket with the Allan Table, for the best specimen of Continuous Gum Work. C. S. Archer, Ind.

VIII. A prize by Spencer & Crocker of a Dental Bracket (the How), full plated, to the Junior Class, for the best specimen of Artificial Dentistry—soldered work. Frank Cunningham, Ind.

The number of matriculates for the session was eighty-one.

